# Association between trimester-specific gestational weight gain and childhood obesity at 5 years of age: results from Shanghai obesity cohort

**DOI:** 10.1186/s12887-019-1517-4

**Published:** 2019-05-02

**Authors:** Wenyi Lu, Xi Zhang, Jiang Wu, Xiaomeng Mao, Xiuhua Shen, Qian Chen, Jun Zhang, Lisu Huang, Qingya Tang

**Affiliations:** 1 Department of Clinical Nutrition, Xin Hua Hospital Affiliated to Shanghai Jiao Tong University School of Medicine, Shanghai, 200092 China; 2 Clinical Research Unit, Xin Hua Hospital affiliated to Shanghai Jiao Tong University School of Medicine, Shanghai, 200092 China; 3 Department of Nutrition, Shanghai Jiao Tong University School of Medicine, Shanghai, 200025 China; 4 Ministry of Education-Shanghai Key Laboratory of Children’s Environmental Health, Xinhua hospital, Shanghai Jiao Tong University School of Medicine, Shanghai, 200092 China; 5 The Department of Pediatrics, Xinhua Hospital, Shanghai Jiao Tong University School of Medicine, Shanghai, 200092 China

**Keywords:** Trimester-specific gestational weight gain, Childhood obesity, Body-fat compositions

## Abstract

**Background:**

It is still unclear if and at which trimester gestational weight gain is related to childhood adiposity. Thus we aimed to evaluate the association between trimester-specific gestational weight gain and body-fat compositions in Chinese children.

**Methods:**

Maternal gestational weight were measured by trained nurses every 2 to 4 weeks from the first prenatal care, and body-fat compositions of 407 children from the Shanghai Obesity Cohort at 5 years of age were measured by nutritionist through bioelectrical impedance analysis. Overweight/obesity of children was defined according to the criteria of International Obesity Task Force. Logistic and linear regression models adjusted for potential confounders were conducted to evaluate the associations of gestational weight gains with childhood obesity and body-fat compositions. Two-sided *P*-value < 0.05 was considered statistically significant.

**Results:**

Greater gestational weight gain in the 1^st^-trimester was significantly associated with a higher risk of childhood overweight/obesity [OR: 1.40 (95% CI: 1.06, 1.86)], fat mass index [β: 0.25 (95% CI: 0.12, 0.38)], body fat percentage [β: 1.04 (95% CI: 0.43, 1.65)], and waist-to-height ratio [β: 0.005 (95% CI: 0.002, 0.008)]. A positive but nonsignificant association was found between greater 3^rd^-trimester gestational weight gain and a higher risk of offspring overweight/obesity, and we speculated that the association between 2^nd^-trimester gestational weight gain and offspring overweight/obesity is the “U” type.

**Conclusions:**

Weight gain in the first trimester gestation is positively correlated with the risk of childhood overweight/obesity and with body adiposity distributions of children at 5 years of age. Weight gain should be well controlled and monitored from early pregnancy.

**Electronic supplementary material:**

The online version of this article (10.1186/s12887-019-1517-4) contains supplementary material, which is available to authorized users.

## Background

The prevalence of childhood obesity constitutes a global health burden [1, 2]. Approximately 60%~ 80% of obese children remain obese as adults [3–5] and childhood obesity may increase the risk of type 2 diabetes, cardiovascular diseases and other chronic metabolic diseases [6, 7]. Previous studies found that total gestational weight gain (GWG) is positively associated with risk of obesity in childhood, adolescence and even adulthood [8–17]. However, most of these studies only used the total GWG as exposure factor other than trimester-specific GWG [10, 15, 18, 19]. It is difficult to distinguish in which specific stage of gestation weight gain has linked with childhood adiposity. Also, body weight or body mass index (BMI) itself is not an accurate index for fat mass distribution especially in assessing child obesity.

Considering these problems, in this study, we used the data from a prospective birth cohort, Shanghai Obesity Cohort, to investigate the associations between maternal GWG in 3 trimesters and childhood obesity at 5 years of age, including fat mass index (FMI), body fat percentage, and fat-free mass index (FFMI).

## Methods

### Participants

**Fig. 1:**
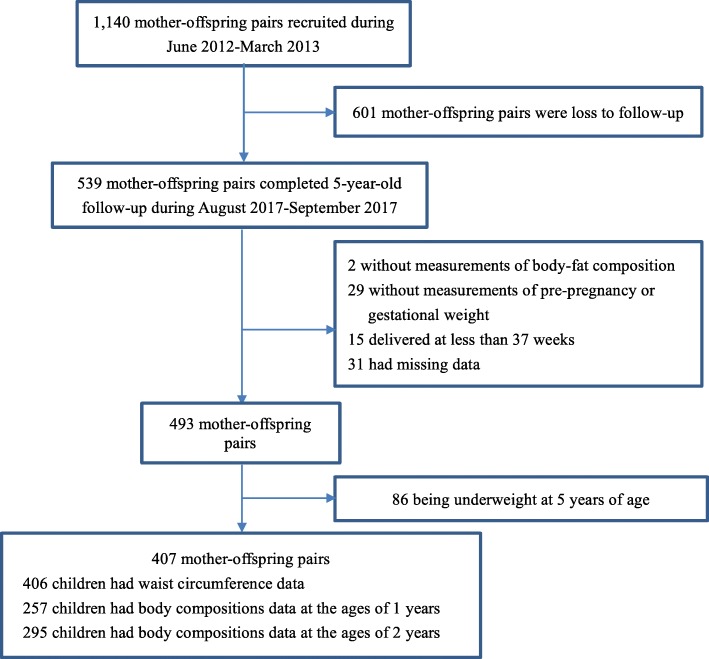
Flow chart for participants selection

Shanghai Obesity Cohort is an ongoing prospective birth cohort. Participants were recruited during June 2012 – March 2013 from two tertiary-level hospitals in Shanghai, Xin Hua Hospital and the International Peace Maternity and Child Health Hospital. Women in the 1^st^-trimester (12–14 gestational weeks) of pregnancy were recruited. Trained research nurses conducted face-to-face interviews with all pregnant women and collected their information on age, education levels, family income and smoking status during pregnancy. Information on maternal weight was also abstracted from hospital electronic records with patients’ consent. We invited all the mother-offspring pairs for the 5-year-old follow-up during August 2017–September 2017. Eventually 539 mother-offspring pairs completed the follow-up face to face by nutritionists and pediatricians at children’s 5 years of age. We excluded 2 pairs without body-fat compositions measurements, 29 pairs without pre-pregnancy weight or gestational weight measurements, and 15 pairs who delivered at less than 37 weeks. There was no significant difference in maternal and children’s characteristics between 31 excluded pairs who had missing data and included pairs, except for duration of breastfeeding (Additional file 1). Then we further excluded 86 pairs being underweight at 5 years of age to reduce the bias in logistic and linear regression models analysis (Fig. 1). Among the remaining 407 pairs, 406 children had waist circumference data, and 257 and 295 children had body compositions data at the ages of 1 and 2 years, respectively.

The ethics approval was obtained from the Institutional Review Broad of Xin Hua Hospital and International Peace Maternity and Infant Health Hospital separately. A written consent was obtained from each participant prior to enter into the study. And parental consent has been obtained from the participants age 16 years below.

### Measurements of gestational weight

Pre-pregnancy weight and height were self-reported and registered at the 1^st^-trimester. Maternal gestational weight was measured at antenatal clinics at each visit by nurses (TCS-150, China). Median number of repeat measurements per woman: 10, interquartile range: 3. Since mothers may visit at variable gestational day, linear interpolation was applied to calculate GWG for the 1^st^ and 2^nd^ trimesters [9, 20]. To evaluate the reliability of the calculated GWG, we conducted a study comparing calculated with clinically measured weight at gestational 13 weeks and 27 weeks. And we found that calculated weight was linearly correlated with clinically measured weight both at gestational 13 weeks and 27 weeks. The Pearson’s correlation coefficient is 0.989 for gestational 13 weeks and 0.994 for 27 weeks, respectively [9, 21, 22]. We calculated the weight gain in the 1^st^ trimester as the weight difference between the pre-pregnancy and 13 gestational weeks, weight gain in the 2^nd^-trimester as the weigh changes from 13 weeks to 27 weeks, weight gain in the 3^rd^-trimester as changes from 27 weeks to the day of delivery. Maternal pre-pregnancy BMI and paternal BMI were categorized as underweight (< 18.5 kg/m^2^), normal weight (18.5–23.9 kg/m^2^), overweight (24.0–27.9 kg/m^2^), or obese (≥ 28.0 kg/m^2^) according to the Chinese BMI Classification [23].

### Measurements of offspring body-fat compositions

Offspring weight and length were measured by trained nurses at birth, 1, 2 and 5 years of age using Seca 416/Seca 217 (Germany) and Tanita 585 (Japan). Considering the difference between each child, we calculated the fat mass index (FMI) and the fat-free mass index (FFMI) using the offspring fat mass and fat-free mass measured by trained nutritionist through bioelectrical impedance analysis (InBody 720, Biospace, South Korea) at 5 years of age [24]. Percentage body fat was also obtained through bioelectrical impedance analysis. We used the waist-to-height ratio (WHtR) to evaluate the abdominal obesity in children. Waist circumference was measured at 5 years of age using a measuring tape placed 1 cm above the umbilicus; it was measured to the nearest 0.1 cm with the child in a standing position. We calculated the BMI and converted raw values into age- and sex-specific standard deviation (SD) scores using World Health Organization reference data [25]. Infant large for gestational age (LGA) was considered a birthweight above the 90th centile for gestational age by 2013 Fenton [26]. Childhood overweight/obesity was defined according to the age- and sex-specific unofficial Asian BMI cut-off points proposed by International Obesity Task Force [27].

### Measurements of covariates

Maternal and paternal information, including maternal education years (< 9 years, 9–11 years, or ≥ 12 years), history of gestational diabetes (yes or no), antibiotic use during pregnancy (yes or no), smoking during pregnancy (yes or no), height (m), pre-pregnancy weight (kg), paternal weight (kg) and height (m), and annual family income (< $15,650, $15,650~$31,300, ≥ $31,300, or refusal to answer), were collected via a face-to-face interview. Research assistants abstracted information on maternal age, parity, mode of delivery (caesarean section, or vaginal), infants’ sex, birth weight (kg), body length (cm), and gestational age from medical records. The information on duration of breastfeeding (< 6 months or ≥ 6 months) was collected via telephone interview when the child was 6 months old.

### Statistical analyses

The characteristics of mothers and children are presented as the means ± SDs or medians (Interquartile ranges). Comparisons among characteristics of mothers and children based on childhood overweight/obesity were conducted using One-way ANOVA and chi-square tests. In order to reduce the bias, we excluded 86 pairs being underweight at 5 years of age in logistic and linear regression models analysis. Because the average SD of trimester-specific GWG was 3 kg, we used logistic regression models to calculate the odds ratio (OR) of LGA and childhood overweight/obesity for each 3 kg increment in gestational weight in trimester-specific gestational. We used linear regression models to examine the relations between GWG and childhood body-fat compositions, including FMI, percent fat, FFMI, WHtR and BMI SD-scores. Variables included in model 1 were the maternal gestational age, education, history of gestaional diabetes, antibiotic use during pregnancy, smoking during pregnancy, family income, parity, mode of delivery, and infant sex. In the model 2, we additionally adjusted for maternal pre-pregnancy BMI, paternal BMI and duration of breastfeeding. Predicted probabilities for offspring overweight/obesity according Model 2 were calculated. After stratified by maternal pre-pregnancy BMI, we used logistic regression models to calculate the ORs of childhood overweight/obesity, and linear regression models to assess the associations between maternal GWG and childhood body-fat compositions. All the models of the stratification analyses were adjusted for maternal education, age, parity, smoking during pregnancy, annual family income, history of gestational diabetes mellitus and antibiotic use, mode of delivery, paternal BMI, offspring sex, and duration of breastfeeding.

All analyses were performed using IBM SPSS Statistics version 25 (IBM Corp., Armonk, NY, USA) and Stata version 14.0 (StataCorp, College Station, TX, USA). Two-sided *P*-value < 0.05 was considered statistically significant.

## Results

Age of maternal gestation ranged from 21 to 50 years, with a median of 29 years (SD: 3). The median birth weight was 3444 g (SD: 418), and 6.7% of them were LGA. At 5 years of age, we observed 17.2 and 7.3% of children becoming to be overweight and obesity, respectively. The rates of gestational diabetes, antibiotic use, and smoking in pregnancy was similar between mothers of normal-weight and obese children (Table 1). For those obese children, their mothers generally had higher pre-pregnancy BMI and lower annual family incomes, the children themselves also had relative high birth weights, BMI at 1 and 2 years of age (Table 1). The median GWG were 2.56 kg (SD: 3.28) in the 1^st^-trimester, 7.04 kg (SD: 2.57) in the 2^nd^- trimester and 6.81 kg (SD: 2.73) in the 3^rd^-trimester gestation.

**Table 1:** Maternal and children’s characteristics in underweight, normal weight and overweight/obesity children

Characteristics	Underweight	Normal weight	Overweight/obesity	*P* value
	*n* = 86	*n* = 286	*n* = 121	
Maternal characteristics				
Pre-pregnancy BMI (kg/m^2^)				**< 0.001**
< 18.5	19 (22.1)	58 (20.3)	10 (8.3)	
18.5–23.9	60 (69.8)	175 (61.2)	75 (62)	
24–27.9	7 (8.1)	45 (15.7)	23 (19)	
≥ 28	0 (0)	8 (2.8)	13 (10.7)	
Age at delivery (years)	29 ± 4	29.0 ± 4	28.0 ± 4	0.467
Education years				0.079
< 9	0 (0)	4 (1.4)	5 (4.1)	
9–12	5 (5.8)	35 (12.2)	10 (8.3)	
≥ 12	81 (94.2)	247 (86.4)	106 (87.6)	
Gestational diabetes mellitus	11 (12.8)	30 (10.5)	17 (14)	0.564
Antibiotic use in pregnancy	2 (2.3)	12 (4.2)	3 (2.5)	0.659
Smoking in pregnancy	0 (0)	1 (0.3)	0 (0)	1.000
Annual family income				**0.045**
< $15,650	23 (26.7)	76 (26.6)	36 (29.8)	
$15,650-31,300	34 (39.5)	95 (33.2)	55 (45.5)	
≥ $31,300	16 (18.6)	54 (18.9)	9 (7.4)	
Refuse to answer	13 (15.1)	61 (21.3)	21 (17.4)	
Mode of delivery				0.324
Vaginal	28 (32.6)	78 (27.3)	28 (23.1)	
Caesarean section	58 (67.4)	208 (72.7)	93 (76.9)	
Primipara	75 (87.2)	249 (87.1)	109 (90.1)	0.683
Duration of breastfeeding ≥6 months (*n* = 357)	52 (92.9)	187 (89.5)	80 (87)	0.527
Paternal BMI (kg/m^2^)				**< 0.001**
< 18.5	6 (7.0)	7 (2.4)	0 (0)	
18.5–23.9	48 (55.8)	139 (48.6)	39 (32.2)	
24–27.9	26 (30.2)	111 (38.8)	53 (43.8)	
≥ 28	6 (7.0)	29 (10.1)	29 (24)	
Total GWG (kg)	15.7 ± 4.4	16.2 ± 5.8	17.3 ± 5.6	**0.048**
1^st^-trimester GWG (kg)	2.4 ± 2.3	2.4 ± 3.4	3.1 ± 3.6	0.130
2^nd^-trimester GWG (kg)	6.8 ± 2.3	7.1 ± 2.4	7.0 ± 3.1	0.572
3^rd^-trimester GWG (kg)	6.6 ± 2.6	6.7 ± 2.7	7.2 ± 2.8	0.263
Children’s characteristics				
Boys	40 (46.5)	139 (48.6)	72 (59.5)	0.088
Birth weight (kg)	3267 ± 355	3446 ± 410	3564 ± 437	**< 0.001**
LGA	0 (0)	20 (7)	13 (10.7)	**0.009**
BMI at 1 year of age (kg/m^2^) (*n* = 308)	16.4 ± 1.0	17.3 ± 1.5	17.9 ± 2.2	**< 0.001**
BMI at 2 years of age (kg/m^2^) (*n* = 355)	15.0 ± 0.9	16.0 ± 1.3	17.0 ± 1.4	**< 0.001**
BMI at 5 years of age (kg/m^2^)	13.6 ± 0.4	15.3 ± 1.0	17.6 ± 1.7	**< 0.001**
FMI at 5 years of age (kg/m^2^)	1.6 ± 0.5	2.4 ± 1.0	4.0 ± 1.6	**< 0.001**
Body fat percentage at 5 years of age (%)	12.0 ± 3.5	16.2 ± 4.4	22.7 ± 6.7	**< 0.001**
FFMI at 5 years of age (kg/m^2^)	12.0 ± 0.5	12.8 ± 0.7	13.5 ± 1.1	**< 0.001**
Waist-height-ratio (*n* = 490)	0.44 ± 0.02	0.46 ± 0.03	0.51 ± 0.04	**< 0.001**

*BMI* body mass index, *GWG* gestational weight gain, *LGA* large for gestational age, *FMI* fat mass index, *FFMI* fat free mass index, *WHtR* waist-height-ratio

### Trimester-specific GWG and childhood overweight/obesity

The multiple regression model 1 and 2 (Table 2) found that a greater 1^st^-trimester GWG was associated with higher offspring BMI SD-scores at 2 years of age [β: 0.08 (95% CI: 0.01, 0.15), β: 0.12 (95% CI: 0.05, 0.19), respectively] and at 5 years of age [β: 0.09 (95% CI: 0.03, 0.15), β: 0.13 (95% CI: 0.07,0.19), respectively]. Each 3 kg increase in 1^st^-trimester GWG associated with 1.40 increment in the risk of overweight/obesity at 5 years of age [OR: 1.25 (95% CI: 1.004, 1.55), OR: 1.40 (95% CI: 1.06, 1.86), respectively]. Greater 1^st^-trimester GWG was also significantly associated with higher FMI [β: 0.16 (95% CI: 0.05, 0.28), β: 0.25(95% CI: 0.12, 0.38), respectively] and body fat percentage [β: 0.68 (95% CI: 0.14, 1.22), β: 1.04 (95% CI: 0.43, 1.65), respectively].

**Table 2:** Associations between trimester-specific GWG and childhood body-fat compositions

Outcomes		Gestational weight gain (per 3 kg)							
		Model 1^a^				Model 2^b^			
		n	1^st^-trimester	2^nd^-trimester	3^rd^-trimester	n	1^st^-trimester	2^nd^-trimester	3^rd^-trimester
Birth to 2 years of age									
LGA	OR (95% CI)	407	1.23 (0.87, 1.74)	**2.40 (1.49, 3.87)**	1.45 (0.98, 2.15)	407	1.22 (0.87, 1.72)	**2.54 (1.56, 4.14)**	1.44 (0.97, 2.15)
BMI SD-scores at 1 year of age	β (95% CI)	257	0.05(−0.02, 0.12)	0.03(− 0.07, 0.13)	− 0.03(− 0.12, 0.06)	231	0.07(− 0.002, 0.15)	0.01(− 0.02, 0.03)	− 0.02(− 0.12, 0.08)
BMI SD-scores at 2 years of age	β (95% CI)	295	**0.08 (0.01, 0.15)**	0.07(− 0.02, 0.16)	− 0.01(− 0.09, 0.08)	245	**0.12 (0.05, 0.19)**	0.06(− 0.03, 0.17)	− 0.02(− 0.12, 0.08)
At 5 years of age									
Overweight/obesity	OR (95% CI)	407	**1.25 (1.004, 1.55)**	0.94 (0.72, 1.21)	**1.33 (1.03, 1.71)**	301	**1.40 (1.06, 1.86)**	1.01 (0.76, 1.48)	1.24 (0.90, 1.70)
BMI SD-scores	β (95% CI)	407	**0.09 (0.03, 0.15)**	−0.0001(− 0.07, 0.07)	0.06(− 0.01, 0.13)	301	**0.13 (0.07, 0.19)**	0.05(− 0.04, 0.13)	0.04(− 0.04, 0.13)
FMI (kg/m^2^)	β (95% CI)	407	**0.16 (0.05, 0.28)**	0.001(−0.15, 0.15)	0.14(− 0.01, 0.28)	301	**0.25 (0.12, 0.38)**	0.11(− 0.07, 0.29)	0.08(− 0.09, 0.25)
Body fat percentage (%)	β (95% CI)	407	**0.68 (0.14, 1.22)**	0.10(−0.61, 0.81)	0.68(− 0.01, 1.36)	301	**1.04 (0.43, 1.65)**	0.51(−0.34, 1.36)	0.47(− 0.31, 1.26)
FFMI (kg/m^2^)	β (95% CI)	407	0.04(−0.02, 0.11)	−0.03(− 0.12, 0.06)	0.02(− 0.07, 0.11)	301	0.05(− 0.03, 0.13)	− 0.02(− 0.13, 0.09)	−0.01(− 0.11, 0.10)
WHtR	β (95% CI)	406	**0.004 (0.001, 0.007)**	−0.002(− 0.005, 0.002)	0.001(− 0.002, 0.005)	300	**0.005 (0.002, 0.008)**	0.0003(− 0.005, 0.004)	0.001(− 0.003, 0.005)

*LGA* large for gestational age, *BMI* body mass index, *FMI* fat mass index, *FFMI* fat free mass index, *WHtR* waist-height-ratio, *OR* odds ratio

^a^ Model 1: adjusted for maternal education, age, parity, smoking during pregnancy, annual family income, history of gestational diabetes mellitus and antibiotic use, and offspring sex; all anthropometry outcomes other than LGA were adjusted for mode of delivery

^b^ Model 2: additionally adjusted for maternal pre-pregnancy BMI and paternal BMI; for all models of body-fat compositions other than LGA were adjusted for duration of breastfeeding. Significant associations are in bold

Greater 2^nd^-trimester GWG was significantly associated with a higher risk of LGA [OR of model 1:2.40 (95% CI: 1.49, 3.87), OR of model 2: 2.54 (95% CI: 1.56, 4.14)]. While greater 3^rd^-trimester GWG was significantly associated with a higher risk of overweight/obesity at 5 years of age [OR:1.33 (95% CI: 1.03, 1.71)] only in model 1.

**Fig. 2:**
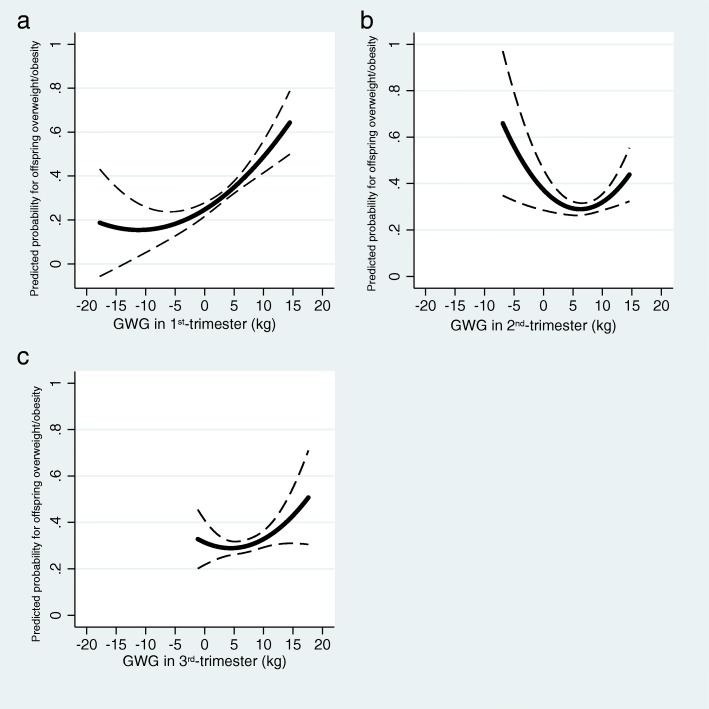
Non-linearity associations between predicted probabilities of childhood overweight/obesity and trimester-specific GWG (kg). Predicted probability and 95% CIs for offspring overweight/obesity by GWG (kg) in (**a**) 1^st^-, (**b**) 2^nd^- and (**c**) 3^rd^-trimester at 5 years of age after adjusting for maternal pre-pregnancy BMI, education, age, parity, smoking during pregnancy, annual family income, history of gestational diabetes mellitus and antibiotic use, mode of delivery, paternal BMI, offspring sex, and duration of breastfeeding. GWG, gestational weight gain; BMI, body mass index

After we draw a graph (Fig. 2) with predicted probability and 95% confidence intervals for offspring overweight/obesity by trimester-specific GWG, we found a positive but nonsignificant association between greater 3^rd^-trimester GWG and a higher risk of offspring overweight/obesity. And we speculated that the association between 2^nd^-trimester GWG and offspring overweight/obesity is the “U” type (Fig. 2). This needs to be confirmed by further large sample studies.

### Stratification analyses by pre-pregnancy BMI status

In women with normal weight before getting pregnant (Table 3), greater 1^st^-trimester GWG was associated with higher childhood FMI [β: 0.27 (95% CI: 0.08, 0.47)] and body fat percentage [β:1.36 (95% CI:0.37, 2.36)]. 2^nd^-trimester GWG had no association with childhood body-fat compositions. In women with overweight/obesity before pregnancy, there was a positive association between 3^rd^-trimester GWG and risk of childhood overweight/obesity [OR:2.76 (95% CI: 1.16, 6.55)] and WHtR [β:0.01 (95% CI:0.00008, 0.03)], even if we further adjusted for duration of breastfeeding, maternal pre-pregnancy BMI, and potential BMI.

**Table 3:** Stratified associations between trimester-specific GWG and offspring body-fat compositions at 5 years of age by pre-pregnancy BMI categories

Outcomes		Pre-pregnancy BMI	n	Gestational weight gain (per 3 kg)		
				1^st^ trimester	2^nd^ trimester	3^rd^ trimester
Overweight/obesity	OR (95% CI)	underweight	50	1.06 (0.28, 3.96)	0.90 (0.19, 4.24)	1.04 (0.17, 6.20)
		normal weight	180	1.33 (0.88, 2.01)	1.09 (0.67, 1.77)	1.18 (0.77, 1.83)
		overweight/obese	71	1.29 (0.79, 2.12)	0.99 (0.55, 1.79)	**2.76 (1.16, 6.55)**
FMI (kg/m^2^)	β (95% CI)	underweight	50	−0.08(−0.31, 0.16)	−0.04(− 0.33, 0.26)	0.19(− 0.09, 0.46)
		normal weight	180	**0.27 (0.08, 0.47)**	0.20(−0.05, 0.44)	0.15(−0.07, 0.36)
		overweight/obese	71	0.19(−0.07, 0.45)	0.28(−0.15, 0.71)	0.50(− 0.03, 1.02)
Body fat percentage (%)	β (95% CI)	underweight	50	−0.55(−1.93, 0.83)	− 0.33(−2.03, 1.37)	1.05(− 0.54, 2.63)
		normal weight	180	**1.36 (0.37, 2.36)**	0.95(−0.28, 2.18)	0.83(−0.26, 1.93)
		overweight/obese	71	0.74(−0.32, 1.79)	1.06(−0.70, 2.82)	2.02(− 0.12, 4.17)
FFMI (kg/m^2^)	β (95% CI)	underweight	50	0.08(−0.21, 0.36)	0.13(−0.22, 0.47)	0.003(− 0.33, 0.34)
		normal weight	180	−0.01(− 0.15, 0.13)	0.01(− 0.16, 0.18)	−0.05(− 0.20, 0.10)
		overweight/obese	71	0.07(−0.04, 0.18)	−0.01(− 0.20, 0.18)	0.10(− 0.13, 0.34)
WHtR	β (95% CI)	underweight	50	0.002(−0.01, 0.01)	0.008(−0.003, 0.02)	0.01(− 0.001, 0.02)
		normal weight	179	0.004(−0.001, 0.01)	0.0001(−0.006, 0.007)	0.001(− 0.004, 0.007)
		overweight/obese	71	0.003(−0.003, 0.009)	0.003(−0.007, 0.013)	**0.01 (0.00008, 0.03)**

*BMI* body mass index, *FMI* fat mass index, *FFMI* fat free mass index, *WHtR* waist-height-ratio, *OR* odds ratio

All models were adjusted for maternal, education, age, parity, smoking during pregnancy, annual family income, history of gestational diabetes mellitus and antibiotic use, mode of delivery, paternal BMI, offspring sex, and duration of breastfeeding. Significant associations are in bold

## Discussion

Our results suggested that larger 1^st^-trimester GWG was positively associated with higher BMI SD-scores at 2 and 5 years of age, and a higher risk of childhood overweight/obesity at 5 years of age. And we found a positive but nonsignificant association between greater 3^rd^-trimester GWG and a higher risk of offspring overweight/obesity. After stratified by pre-pregnancy BMI status, we found that greater 3^rd^-trimester GWG in women with pre-pregnancy overweigh/obesity was associated with higher risk of offspring overweight/obesity.

Our finding about 1^st^-trimester GWG was consistent with data from previous cohort studies [8–10, 12–15], which used BMI as indicators to evaluate offspring obesity. About 3^rd^-trimester GWG, only one previous study found that GWG of more than 500 g/per week after 14-week gestation was associated with a higher offspring BMI and waist circumference [10]. Another animal model showed that the increased nutrient supply in late gestation was associated with fetus leptin synthesis, fat deposition, and circulating leptin concentrations [28]. In this study, we extend previous knowledge by measuring children’s body-fat compositions using bioelectrical impedance analysis, which had no radiation and costs less than dual energy X-ray absorptiometry. We found childhood FMI, body fat percentage and abdominal obesity were positively associated with maternal GWG at 1^st^-trimester. After stratified by pre-pregnancy BMI status, the maternal GWG-children’s adiposity association was remained only among normal weight women. This finding was similar to one previous report, which showed a positive association between greater 1^st^-trimester GWG and offspring FMI in pregnant women with pre-pregnancy normal weight and obesity [9]. Since we merged women with pre-pregnancy overweight and obesity into one group in the stratified analysis, this may have led to an inconsistent result for this group.

Several mechanism hypotheses could explain the relationship between 1^st^-trimester GWG and childhood obesity. First, animal studies have suggested that overnutrition during pregnancy will stimulate the proliferation of neurons expressing orexigenic peptides in the hypothalamus [29]. In addition, maternal consumption of a high-energy diet during pregnancy can affect the sensitivity of the offspring’s ventromedial hypothalamic nucleus neurons to glucose and long-chain fatty acids [30]. The increase of blood glucose and plasma free fatty acids in pregnant women with obesity may increase the transmission of nutrients to the placenta during embryonic and fetal growth. This results in changes in appetite control, neuroendocrine functioning, or energy metabolism in the developing fetus [31]. These changes may be among the factors responsible for offspring obesity. In addition, early pregnancy is an important period for the development of the central nervous system, including the hypothalamus. Secondly, compared with 2^nd^-and 3^rd^-trimester GWG, which is more strongly affected by placental and fetal growth, 1^st^-trimester GWG is more reflective of maternal weight and adiposity deposition [32, 33]. The greater the maternal weight and adiposity deposition, the greater indication that the mother might prefer a lifestyle characterized by a high-energy diet and low physical activity. One previous study suggested that greater 1^st^-trimester GWG was associated with more maternal weight gain and a higher waist circumference at 3 and 7 years postpartum [20]. These mothers’ offspring might share these lifestyle habits. Thirdly, a study of 88 mother-offspring pairs suggested that greater GWG in the early pregnancy (within 18 weeks) was associated with higher DNA methylation in the offspring’s cord blood [34]. The obese maternal metabolic environment affects early placental growth and gene expression, including mitochondrial dysfunction, decreased energy metabolism and expression of leptin receptor [35, 36]. Lower energy expenditure and higher energy intake was observed in infants born to overweight mothers compared with lean mothers at 3 months [37].

Previous studies have shown no consistent finding regarding on the linkage between 2^nd^-trimester GWG and childhood obesity. Only one study found a significant positive association between greater 2^nd^-trimester GWG and higher offspring BMI at 7 years of age [14]. Another study showed that greater 2^nd^ trimester GWG rate was associated with a higher risk of offspring overweight, but not with a higher risk of obesity [15]. While Hivert’s study showed that greater 2^nd^-trimester GWG was not only associated with higher risk of childhood obesity and a higher BMI SD-score, and also a higher FMI, FFMI and waist circumference [9]. Fraser’s study showed that high offspring BMI, waist circumference and FM were observed in children born to mothers whose GWG was above 500 g/per week between 14- and 36-week gestation [10]. However, several studies did not find a significant association between 2^nd^-trimester GWG and children’s obesity [8, 13], which was similar with our study. We only found that greater 2^nd^-trimester GWG was associated with higher risk of LGA, which was similar to the previous studies [11, 13, 38].

Our study has some limitations. First, there was a considerable loss to follow-up during the long study period, which would have resulted in bias, especially in the stratification analysis. Second, the pregnant women weight was not measured in same gestational week and the pre-pregnancy weight was self-reported. Although some studies concluded that self-reported maternal gestational weight has a high correlation with weight measurement [39, 40], women generally underestimate their own weight [41], which may lead to a higher GWG than the actual value. And the association between GWG and childhood overweight/obesity would be enhanced. Third, we measured offspring adiposity indices using bioelectrical impedance analysis rather than the gold standard-dual energy X-ray absorptiometry.

## Conclusions

The results of our study suggested that the timing of maternal GWG was critical for offspring childhood obesity. We found that pregnant women should pay more attention to 1^st^-trimester GWG, even if their weight is normal before pregnancy. It is necessary to control the range of 1^st^-trimester GWG. And we speculated that the association between 2^nd^-trimester GWG and offspring overweight/obesity is the “U” type other than a linear association. Further studies with larger samples are needed to confirm our findings.

## Additional file

Additional file 1: Maternal and children’s characteristics in excluded pairs with missing data and included pairs. BMI, body mass index; FMI, fat mass index; FFMI, fat free mass index; WHtR, waist-height-ratio. (XLSX 34 kb)

## Abbreviations

BMI: Body mass index
FFMI: Fat-free mass index
FMI: Fat mass index
GWG: Gestational weight gain
LGA: Large for gestational age
SD: Standard deviation; OR, odds ratio
WHtR: Waist-to-height ratio
